# Stronger perceptual filling-in of spatiotemporal information in the blind spot compared with artificial gaps

**DOI:** 10.1167/jov.20.4.20

**Published:** 2020-04-28

**Authors:** Yulia Revina, Gerrit W. Maus

**Affiliations:** Psychology Programme, School of Social Sciences, Nanyang Technological University, Singapore

**Keywords:** artificial scotomata, blind spot, perceptual filling-in

## Abstract

Complete visual information about a scene and the objects within it is often not available to us. For example, objects may be partly occluded by other objects or have sections missing. In the retinal blind spot, there are no photoreceptors and visual input is not detected. However, owing to perceptual filling-in by the visual system we often do not perceive these gaps. There is a lack of consensus on how much of the mechanism for perceptual filling-in is similar in the case of a natural scotoma, such as the blind spot, and artificial scotomata, such as sections of the stimulus being physically removed. Part of the difficulty in assessing this relationship arises from a lack of direct comparisons between the two cases, with artificial scotomata being tested in different locations in the visual field compared with the blind spot. The peripheral location of the blind spot may explain its enhanced filling-in compared with artificial scotomata, as reported in previous studies. In the present study, we directly compared perceptual filling-in of spatiotemporal information in the blind spot and artificial gaps of the same size and eccentricity. We found stronger perceptual filling-in in the blind spot, suggesting improved filling-in for the blind spot reported in previous studies cannot be simply attributed to its peripheral location.

## Introduction

In the natural world, complete visual information about the scene and the objects within it is often not available to us. For example, objects may be partly occluded by other objects, be under shadow, or have sections missing. Perhaps the visual input might not be detected at all by the retina, as is the case with the blind spot region in which the optic nerve exits the eye and there are no photoreceptors. Nevertheless, in most cases we do not perceive a world riddled with “gaps.” We often have no trouble identifying objects even from partial information (Johnson & Olshausen, 2005; Tang, Buia, Madhavan, Crone, Madsen, Anderson, & Kreiman, 2014), and, in the case of the blind spot, we rarely notice its existence. The visual system allows this seamless perceptual experience by filling-in the missing information. Completion processes can be subdivided into two types: amodal and modal. In amodal completion, the observer infers that a whole object exists even when a part of it is not currently visible. For example, they may see a dog behind a picket fence, but do not perceive the separate visible parts of the dog as individual objects. In modal completion, however, the observer would report visually perceiving features of the scene, which have no retinal counterpart. Such *perceptual* filling-in can occur under various circumstances, such as illusory contours of Kanizsa shapes (Kanizsa, 1976), neon color spreading (Bressan, Mingolla, Spillmann, & Watanabe, 1997), Troxler fading (Troxler, 1804), filling-in in the blind spot (Chen, Maus, Whitney, & Denison, 2017; Durgin, Tripathy, & Levi, 1995; Fiorani Júnior, Rosa, Gattass, & Rocha-Miranda, 1992; Maus & Whitney, 2016; Maus & Nijhawan, 2008; Ramachandran, 1992) and in artificial (Fiorani Júnior et al., 1992; Mendola, Conner, Sharma, Bahekar, & Lemieux, 2006; Meng, Remus, & Tong, 2005; Ramachandran & Gregory, 1991; Spillmann & Kurtenbach, 1992; Tynan & Sekuler, 1975; De Weerd, Gattass, Desimone, & Ungerleider, 1995; Weil, Kilner, Haynes, & Rees, 2007) or pathological (Cohen, Lamarque, Saucet, Provent, Langram, & LeGargasson, 2003; Gerrits & Timmerman, 1969; Ramachandran, 1993; Zur & Ullman, 2003) scotomata.

In the blind spot, perceptual filling-in occurs when spatiotemporally coherent elements are visible on opposite sides (Fiorani Júnior et al., 1992) or around the blind spot (Spillmann, Otte, Hamburger, & Magnussen, 2006). However, an object on one side moving into the blind spot can also be extrapolated under certain conditions (Maus & Whitney, 2016; Maus & Nijhawan, 2008). In addition, Maus and Whitney (2016) showed that moving spatial patterns (sinusoidal gratings) can have their spatiotemporal structure, as opposed to just luminance, filled in across the blind spot to a greater degree than static ones. Color and texture can be filled in across the blind spot as well (Chen et al., 2017; Li, Luo, Lu, Kan, Spillmann, & Wang, 2014; Spillmann et al., 2006).

Perceptual filling-in at the blind spot and other cases of modal, or even amodal, filling-in share some similarities but nevertheless have some differences. In the following paragraphs we review studies on filling-in in the blind spot and artificial or pathological scotomata. We take a broad definition of artificial scotomata to mean any gaps in the stimulus not caused by an anatomic reason (such as blind spot or retinal damage), which can include “patches differing from the surround in brightness, color or texture” (Spillmann, 2011). There is still no clear consensus on how much of the mechanism for filling-in is shared among these different cases of perceptual completion.

Some authors have argued that filling-in at the blind spot uses a different mechanism compared with filling-in in other circumstances. For example, the latency to perceive complete filling-in at the blind spot is almost instantaneous, whereas filling-in across artificial scotomata can take several seconds (Gerrits & Vendrik, 1970; De Weerd, Desimone, & Ungerleider, 1998). Other authors have argued for a different mechanism because of the type of stimuli that can or cannot be filled in across the blind spot. For example, illusory contours might not pass through the blind spot (Maertens & Pollmann, 2007) but can go through pathological scotomata caused by macular degeneration (De Stefani, Pinello, Campana, Mazzarolo, Lo Giudice, & Casco, 2011). Crossland and Bex (2009) found that performance in a Vernier alignment task is better over the blind spot compared with intact temporal retina, which itself was not different from alignment thresholds over a pathological retinal scotoma, suggesting that filling-in across the blind spot is different compared with either artificial or pathological scotomata. Baek, Cha and Chong (2012) found that traveling waves of binocular rivalry dominance propagate through the blind spot, but not an artificial gap of the same size in the intact retina. Results from experiments designed to find the cortical locus of perceptual filling-in also suggest potential differences. For example, filling-in for the blind spot seems to occur as early as V1 (Fiorani Júnior et al., 1992; Komatsu, 2011; Tong & Engel, 2001), whereas other cases of modal completion, such as illusory contours, occur in V2 and later, (Peterhans & von der Heydt, 1989; von der Heydt, Peterhans, & Baumgartner, 1984), again suggesting perceptual filling-in at the blind spot may use a different mechanism compared with other illusory percepts.

However, there is some evidence for a shared mechanism as well. For example, Fiorani Júnior et al. (1992) recorded V1 neurons in monkeys and reported comparable electrophysiological results for the blind spot and an artificially occluded region. Similar findings have also been reported in humans using functional magnetic resonance imaging. For example, Meng, Remus and Tong (2005) found an increase in V1 activity when an illusory stimulus is perceived across an artificial scotoma. Similarly, using illusory figures from Kanizsa-type inducers, Kok, Bains, van Mourik, Norris, & de Lange (2016) found that cortical feedback activity induced by these illusory shapes led to activation of the deep layers of V1. This suggests filling-in, either at the blind spot or across an artificial gap, may use similar neuronal mechanisms. However, Tong and Engel (2001) found that V1 activity corresponding to a filled-in percept during binocular rivalry at the blind spot was weaker than the activity corresponding to when fellow eye stimulation was dominant. Therefore the mechanisms of filling-in remain unclear.

Durgin, Tripathy, and Levi (1995) argue that filling-in at the blind spot is similar to amodal completion of partly occluded stimuli viewed somewhat peripherally, and differences between the blind spot and occlusion often arise due to foveal viewing of amodally completed stimuli in many experiments. Likewise, Ramachandran (1995) proposes that “it is very unlikely that the visual system has evolved dedicated neural machinery for the specific purpose of filling-in the blind spot.”

Part of the difficulty in assessing the similarity of filling-in across the blind spot and other gaps is the paucity of more direct comparisons between the two cases, with artificial or pathological scotomata usually tested at different eccentricities compared with the blind spot. Researchers typically have no choice of location in the visual field and size when studying pathological scotomata, at least in human participants. It has been shown that filling-in in artificial scotomata improves with increasing eccentricity (De Weerd, Desimone, & Ungerleider, 1998). Therefore strong perceptual filling-in in the blind spot could be mostly because of its peripheral location, or alternatively, as some authors have suggested, filling-in at the blind spot could be different compared with artificial gaps, even at an equivalent size and location.

In the present study therefore we aimed to compare perceptual filling-in in the blind spot and artificial gaps more directly by using a gap region of the same size and eccentricity across all conditions. Among artificial gap conditions, we compared cases of occlusion versus deletion (somewhat like an artificial scotoma). At first glance, these two cases may be predicted to lead to the same perceptual properties, as both present a gap in the stimulus. However, a study by Johnson and Olshausen (2005) has compared object identification speed and accuracy under cases of “occlusion” and “deletion,” and even though the same amount of information was missing, cases of occlusion had an advantage, suggesting there could be differences in processing of occlusion and deletion.

We assessed perceptual filling-in (modal completion) using moving sinusoidal gratings that passed either through the blind spot or through artificial gaps of the same size and eccentricity, presented in the fellow eye. Participants performed a numerosity task as previously used by Maus and Whitney (2016), in which they had to indicate which stimulus on a particular trial had more stripes overall. This task does not require the participants to report filling-in per se, but if filling-in occurs, this would lead to biases in judging the spatiotemporal structure of the stimulus. The strength of this approach is that participants were not required to make a subjective report on whether filling-in occurred or not, and additionally we could test for filling-in of spatiotemporal structure, rather than simply the presence of something in the gap. The comparison stimulus with varied spatial frequency (SF) passing through the blind spot was compared with an intact control stimulus of a fixed density. Any perceptual filling-in of the spatiotemporal information across the gap would lead to an overestimation of the total number of stripes in the stimulus. This measure was used as a proxy for filling-in strength. If filling-in across the blind spot is largely because of its peripheral location, we would expect to find similar filling-in strength for the blind spot and equally eccentric artificial gaps. Alternatively, if strong perceptual filling-in at the blind spot uses a different mechanism, we would expect stronger filling-in for the blind spot compared with artificial scotomata or occluded regions of the same size and location.

In addition to our main research question, we tested to what extent individual differences in the strength of perceptual filling-in are related to individual differences in the strength of voluntary mental imagery. Both filling-in and visual imagery are instances of nonretinal vision—perceiving something that was not a direct consequence of external stimulation. To the best of our knowledge, previous studies have not looked at the relationship between mental imagery and perceptual filling-in. However, several studies have investigated the relationship between mental imagery and nonretinal vision other than filling-in. For example, Grzeczkowski, Clarke, Francis, Mast, and Herzog (2017) found that mental imagery strength only correlated with the strength of the Ponzo illusion out of several visual illusions the authors tested, suggesting illusory percepts are not necessarily related to the same mechanisms as voluntary mental imagery. More vivid mental imagery (as self-reported using the Vividness of Visual Imagery Questionnaire [VVIQ], Marks, 1995; Marks, 1973) has been shown to lead to stronger influence of imagery on subsequent perception in a binocular rivalry paradigm—the imagined pattern was more likely to be dominant (Pearson, Clifford, & Tong, 2008, but see Dijkstra, Hinne, Bosch, & van Gerven, 2019, who found an effect of vividness of the imagined stimulus on a particular trial, but not the VVIQ scores). Other studies have looked at whether mental imagery is related to hallucinations. For example, Shine, Keogh, O'Callaghan, Muller, Lewis, and Pearson (2015) found that stronger mental imagery is associated with more visual hallucinations in Parkinson's disease. In a healthy population, Salge, Pollmann, and Reeder (2019) found a correlation between imagery and pareidolia, suggesting that stronger mental imagery can lead to a greater chance of misperceiving an externally presented stimulus. Furthermore, the size of the primary visual cortex has been linked to differences in both imagery (Bergmann, Genç, Kohler, Singer, & Pearson, 2015) and strength of visual illusions (Schwarzkopf & Rees, 2013; Schwarzkopf, Song, & Rees, 2011), with smaller V1 being associated with stronger imagery and stronger Ebbinghaus and Ponzo illusion perception. This suggests, albeit indirectly, that strength of mental imagery might be associated with the strength of, at least some, illusory percepts. It is not clear how perceptual filling-in may relate to hallucinatory experiences or misperceptions in visual illusions, and therefore we did not have a specific hypothesis on whether perceptual filling-in relates to visual imagery, but to explore this question, participants completed the VVIQ as part of the study.

## Methods

### Participants

Sixty-four participants took part in the study (mean age: 24.41 years, range 18–47; 30 men, 34 women; 42 right-dominant eye, 22 left-dominant eye, one equal left and right dominance). Ocular dominance was determined using a simplified version of the Miles test (Miles, 1929). Participants placed their hands near each other to form a triangular opening between the thumbs and index fingers of both hands. With both eyes open, they centered the opening on a distant object. They then continued looking with only the left or the right eye open. The opened eye for which the distant object remained most in the center of the opening was deemed the dominant eye for that participant. We paid participants S$10 per hour, or alternatively they received credits for participating in the experiment as part of their undergraduate course program. Participants provided informed written consent, and the local institutional review board at Nanyang Technological University approved the experiment (IRB-2015-10-049). We excluded five participants due to poor blind spot measurements and poor performance in an orthogonal attentional task, as described in more detail later. Therefore we report results from 59 participants (mean age: 24.22 years, range 18–47 years; 30 men).

### Apparatus and stimuli

Participants sat 70 cm away from the screen while using a chin rest. They wore occlusion shutter glasses during the experiment and were instructed to always fixate on the white cross that was presented off-center on the screen.

Stimuli were presented on a 21-in. FD Premium Sun Microsystems CRT display with a resolution of 1152 × 864 and a refresh rate of 100 Hz, using MATLAB 2015a (The MathWorks Inc., Natick, MA) and Psychtoolbox-3 (Brainard, 1997; Kleiner, Brainard, & Pelli, 2007; Pelli, 1997).

**Figure 1.**
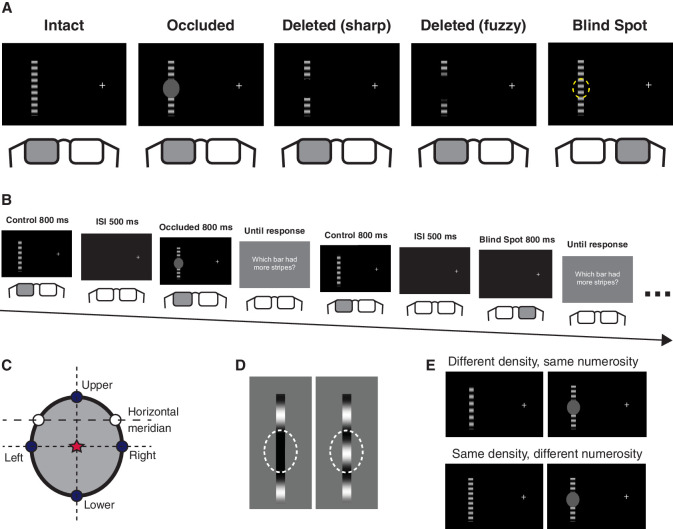
Experimental design. (A) Five stimulus conditions: Intact fully visible bar, Occluded bar with a gray patch covering the middle, Deleted (sharp edge) bar in which a patch was removed from the middle section using a sharp edge, Deleted (fuzzy edge) bar in which a patch was removed from the middle section using a fuzzy edge, Blind Spot in which the Intact bar was viewed through the blind spot. The occluder and the deleted section were the same size as the participant's blind spot and were centered on the blind spot midpoint. Each trial was presented monocularly using shutter glasses. (B) An example experimental sequence showing two trials. The ISI and response screen were viewed binocularly. (C) Blind spot measurement. Each participant's blind spot was estimated using a six-point measurement technique. First, the left and right edges were measured along the horizontal meridian (white dots), followed by the upper and lower points (dark dots) on a trajectory bisecting the initial horizontal diameter, and finally the left and right points (dark dots) were measured again on a trajectory bisecting the vertical diameter. The final blind spot size was determined by an oval centered on the intersection of the vertical and horizontal diameters (red star). (D) Perceptual filling-in of the spatiotemporal information (stripes) through the gap. If no filling-in of the spatiotemporal pattern occurs (and only the homogenous color of the neighboring inducing elements are filled-in), participant's perception will be closer to the left panel. However, if the stripes are completed through the gap, perception will be closer to that shown in the right panel. Adapted from Maus & Whitney (2016). (E) Participants were asked to judge numerosity (number of stripes) and not the density (SF). A stimulus can have different density, but the same numerosity, or the same density but different numerosity.

We used sinusoidal moving gratings of 0.25, 0.30, 0.35, 0.40, or 0.45 cycles/degree (cpd) of visual angle for one group of participants, and gratings of 0.20, 0.30, 0.40, 0.50, 0.60 cpd for another group of participants. Gratings drifted upward at three cycles per second. They were centered on the blind spot, which was measured prior to each block of trials. Each stimulus was presented monocularly using shutter glasses (PLATO Visual Occlusion Spectacles, Translucent Technologies, Toronto, Canada), and the viewing eye was determined by the condition. The five different conditions were (Figure 1A): *Intact* in which the fully visible bar was shown; *Occluded* in which a gray patch covered the middle portion of the bar; *Deleted (sharp edge)* in which a patch was removed from the middle section of the bar with a sharp edge; *Deleted (fuzzy edge)* in which a patch was removed from the middle section of the bar with a fuzzy edge; and *Blind Spot* in which the intact bar was viewed through the blind spot. The occluder and the deleted section were the same size as the participant's blind spot and were centered on the blind spot midpoint. The interstimulus interval (ISI) and the response screen were presented binocularly.

The sinusoidal bars had a maximum brightness of 0.1 x maximum monitor brightness (≈130 cd/m² maximum monitor brightness, ≈13 cd/m^2^ stimulus brightness) and were presented on a black background (≈0.75 cd/m²) with a white fixation cross. Participants sat in a dark room and we presented the stimuli at low luminance to make the switching of the shutter glasses lenses less noticeable for the participants. This reduced any knowledge of the current stimulus condition, even though all participants were naive and were not told which viewing eye corresponded to which condition, or indeed which conditions existed in the experiment. To also minimize dark adaptation, the response screen was mid-gray (≈65 cd/m^2^), as only the monocularly presented stimuli needed to be very dim.

In the Occluded condition, the occluder oval was gray (half of maximum brightness of the grating, ≈6.5 cd/m^2^) and the same size as the blind spot. The occluder outline was a darker gray (0.25 of maximum grating brightness, ≈3.25 cd/m^2^). In the Deleted Sharp condition, a black oval of the same color as the background and the same size as the blind spot was superimposed onto the middle of the bar, to give an effect of a section of the bar being erased in the middle. In the Deleted Fuzzy condition, a two-dimensional (2D) Gaussian mask of the same size (approximately) as the blind spot was superimposed on the bar, to give the impression of an erased section with fuzzy edges. The 2D Gaussian mask was built with an amplitude of 3.5 and standard deviation of just under half the diameter of the blind spot ([blind spot diameter – 5 pixels]/2). This was calculated for the dimension of width and height to achieve an oval Gaussian mask. Values below 0.05 were truncated, and all values above 1 were changed to 1 to give a “flatter” peak.

The sinusoidal grating was 1.73° in width (same as Maus & Whitney, 2016) and blind spot height + 10° vertically. A 5° section of bar was visible above and below the blind spot. Because the blind spot vertical dimension was not the same for each participant, the total length of the bar varied between subjects.

### Procedure

#### Blind spot measurement

Prior to each block, we measured each participant's blind spot using six points (similar to the procedure used in Chen et al., 2017; Maus & Whitney, 2016). During this, the fellow eye was occluded using shutter glasses. Participants moved a flickering cursor (alternating between black and white, 10 × 10 pixels [0.3°] in size) across a gray screen using the computer mouse and indicated the “first point at which it disappeared” via a left mouse click. Participants fixated on a white cross that was offset from the center of the screen and moved the cursor from fixation to either the left or the right to map the inner points for the left and right eye, respectively; from the outer edge of the computer monitor toward the fixation to map the outer points; from the top of the monitor downward to map the upper points; and from the bottom of the monitor upward to map the lower points. Participants were encouraged to take their time and move the cursor back and forth near the edge of the blind spot, to make sure they were measuring the edge as closely as possible. First, the preliminary inner and outer points were measured along the horizontal meridian (Figure 1C, white dots), followed by the upper and lower points on a trajectory bisecting the preliminary horizontal diameter. Finally, the inner and outer points were remeasured on a trajectory bisecting the vertical line. The final blind spot was approximated by a symmetrical oval between the four coordinates (Figure 1C, dark dots) using the midpoint of the horizontal and vertical diameters to determine the horizontal and vertical coordinates of the blind spot center (Figure 1C, red star). Each of the six points was measured three times, and the mean of the three measurements was subsequently used. If the coordinates of the three points were not close together (for example, the participant accidentally measured the upper instead of lower for one of the points), the blind spot was remeasured for that block. After the procedure, the blind spot oval was presented on the screen, and participants were asked to fixate on the cross and verify they do not see the oval when viewing monocularly with the blind spot eye. If the participant could see some parts of the oval, the procedure was repeated to achieve a better estimate of their blind spot size.

#### Main experimental task

The main experiment consisted of four blocks of 250 trials each. Therefore each participant completed 1000 trials in total. Participants could take a break every 50 trials and at the end of each block. There were 500 trials for each blind spot eye. There were 40 trials for each individual stimulus overall (25 unique types of bar: five levels of SF x five conditions, presented 10 times in each block). Figure 1B shows an example of a trial sequence.

On each trial, the control and the comparison stimuli were presented for 800 ms each, separated by a 500 ms ISI. The goggles opened 350 ms after the (programmed) stimulus offset to account for a gradual stimulus fade-out on the monitor to prevent any binocular viewing of a given stimulus. The stimuli were presented offset from the screen center and centered on the previously measured blind spot location. The order of the control and comparison stimuli was counterbalanced for all participants (except eight in the 0.25–0.45 group, for whom the control stimulus appeared first). At the end of the stimuli presentation the participant was asked to indicate whether the first or the second bar had more stripes overall. We emphasized that they should judge the total number of visible stripes in the stimulus, rather than the density of the stripes outside the gap because we were interested in judgments of numerosity rather than SF. Participants pressed the left arrow key on the keyboard to answer *first* and the right arrow key to answer *second.* The trials were self-paced, with the participant pressing the space bar to move on to the next trial after responding.

On a few of the trials (five in each block of 250), there was an additional task (except for the participants in the noncounterbalanced group mentioned earlier). The white fixation cross the participants were instructed to always look at changed to red for one of the stimuli presentations. We kept the additional task rare so as to interfere minimally with the primary task and keep the experiment length reasonable (not having a second key press on every trial). However, participants were encouraged to watch closely for this rare color change to “not miss any.” At the start of the experiment, participants were not allowed to move on to the main set of trials if they missed any fixation changes during the training block. If the participant saw the color change on a given trial, they had to press the down arrow key on the keyboard to indicate this, after the usual response of *first* or *second.* The purpose of this additional task was to discourage any eye movements toward the stimulus bars, as it was important that they were viewed peripherally.

#### Visual imagery measurement

In addition to the experimental blocks, participants completed a version of the VVIQ (Marks, 1995; Marks, 1973). We reversed the scoring so that a score of 1 signified the weakest imagery and a score of 5 the strongest. Participants responded to 16 questions with their eyes open, and the same questions while visualizing with their eyes closed.

### Data analyses

#### Psychometric function plotting

We plotted the proportion of responses in which the participant answered “comparison has more stripes” for each level of SF. We then fitted a psychometric function to the data points with the Palamedes toolbox for MATLAB (Prins & Kingdom, 2018), using the maximum likelihood fitting procedure with the logistic function with the following parameters: gamma (guess rate) = 0.02 and lambda (lapse rate) = 0.02.

#### Points of subjective equality calculation

To estimate greater or lower amounts of perceptual filling-in between conditions, we looked for biases in responding that the comparison stimulus had more stripes. We calculated the points of subjective equality (PSE), which is the SF for which 50% of the participants’ responses were “comparison has more stripes.” In other words, this is the SF at which the comparison stimulus looked the same to the participant as the control stimulus of 0.3 cpd. For the conditions with a gap, we would expect PSEs greater than 0.3 because a higher density of stripes (i.e., a higher SF) is needed to have equivalence to the control bar because of the missing middle patch. Values of PSE closer to 0.3 suggest more stripes being perceived than in conditions with PSEs further away from 0.3 cpd.

#### Points of true equality calculation

To estimate filling-in in each condition we can also compare the number of stripes perceived and the true (veridical) number of stripes in that condition. To do this, we calculated the SF the bar would be required to have in order to be veridically equivalent to the 0.3 cpd control bar. Because of the gap in the middle, the comparison bars need to be of a higher density (higher SF) to be equivalent in the number of stripes overall to the Intact stimulus. We refer to this value as the point of *true* equality (PTE, the cpd at which the gap stimulus should appear equivalent when no perceptual filling-in occurs). We calculated the PTE for each participant separately because although the density of the bar was the same across subjects, the total length of the bar was not. The visible portion of the bar outside the occluder/blind spot was always 10°, but the vertical dimension of the blind spot was different for each participant, thus making the total length of the bar different between subjects. For participants with bigger blind spot vertical sizes, and thus longer Intact stimuli, the PTE for the gap stimuli was higher. If the PSEs were significantly lower than PTEs, this suggests an overestimation of the true number of stripes in the bar, and therefore suggests a certain degree of perceptual filling-in.

#### Participant exclusion criteria

For some participants we obtained flat psychometric functions (they never responded that the comparison stimulus had more stripes, suggesting they never experienced perceptual filling-in), and therefore the PSEs we obtained were very large and outside our tested range. We cannot be sure such PSEs were estimated reliably, and therefore we removed such data points from the statistical analysis. To determine which psychometric functions were poor fits, we ran the goodness of fit procedure in the Palamedes toolbox with 1000 simulations. Functions with a pDev value of < 0.05 were counted as poor fits and were removed. In addition, we looked at the number of functions that converged out of 1000. We removed data points from functions that converged fewer than 950/1000 times and the estimated PSE was outside our tested range (0.25–0.45 for first group, 0.20–0.60 for second group). This targeted flat psychometric functions with unusually large PSEs. Similar to the PSE comparisons, we removed slope values from statistical analysis from psychometric functions that were poor fits (pDev < 0.05). In addition, we removed slope values from functions matching all of the following three criteria: (a) failed to converge on at least 95% of the simulations, (b) PSE outside of the stimulus range, and (c) large slope value labeled as outlier (>1.5 interquartile ranges above the 75th percentile). This removed unusually large slopes for unusually large PSEs, which could not be properly estimated, but kept small slope values corresponding to flat functions. We determined those small slope values representing no filling-in to be meaningful (from participants always responding that the comparison had fewer stripes, leading to flat functions).

#### Statistical testing

We performed a Wilcoxon signed-rank test to examine for significant differences between each pair of conditions. We used a nonparametric test to compare differences between conditions due to a few large PSEs, or small slope values that are skewing the distribution in the conditions with the gap. However, we also calculated parametric statistics (paired *t*-test) to calculate a Bayes factor (*B*) for each comparison with the method described by Dienes (2014) using the author's online calculator. Using this approach, we are able to determine whether nonsignificant results using the more traditional method stem from the null hypothesis being supported, or whether the data are insensitive and do not provide evidence for either hypothesis. A Bayes factor greater than 3 suggests substantial evidence for the alternative hypothesis, whereas a Bayes factor lower than 0.33 suggests substantial evidence for the null hypothesis. Bayes factor values between 0.33 and 3 indicate that the data are insensitive. Data may be insensitive due to high variability, but with sufficient N, the Bayes factor value would support either the null or the alternative hypothesis.

## Results

### Blind spot measurements

The average blind spot size was 5.18° x 6.24° (*SD* = 1.01° x 1.10°) horizontally and vertically, respectively. Its center was located on average at 16.44° (*SD* = 1.05°) eccentricity. This is consistent with previously reported measurements using a similar procedure (Chen et al., 2017; Maus & Whitney, 2016). We identified three participants whose measured blind spot sizes were deemed as outliers (>1.5 interquartile ranges away from 25th and 75th percentiles; 7.94°, 2.90°, and 2.79° in the horizontal width). These participants were excluded from all further analyses.

### Fixation point task performance

Throughout the experimental blocks, participants were asked to fixate on a cross and report when it briefly changed color. This was to encourage good fixation and minimize eye movements disrupting the correspondence of the stimulus location with the blind spot location. The fixation cross changed color from white to red on five trials out of 250 in each block. If participants never responded to the color change, this would be 98% accuracy on the task. If participants always responded that they saw the color change, this would be 2% accuracy. Finally, random responses would yield 50% accuracy. The average fixation task performance was 99.75%. The average participant missed 1.78 color changes and had 0.67 false positives. We removed two participants with less than 99% accuracy from all further analyses (98.5% and 98.8% accuracy).

### Perceptual filling-in is higher for the blind spot than artificial scotomata at the same eccentricity

We estimated the degree of perceptual filling-in using a numerosity judgement task (Maus & Whitney, 2016). Any perceptual filling-in of the spatiotemporal pattern across the gap would lead participants to perceive more stripes overall in that stimulus, and therefore more likely to report the comparison stimulus as having more stripes than the veridical number. We considered this overestimation of the number of stripes in the comparison stimulus as a marker for perceptual filling-in.

We compared perceptual filling-in in the different conditions by comparing shifts in the psychometric functions. We calculated PSE as the SF at which there is 50% probability of reporting the comparison stimulus as having more stripes than the control. The control stimulus was always 0.3 cpd, and therefore an observer with no bias (overestimation or underestimation of the number of stripes) would have a PSE at 0.3. A rightward shift in the psychometric functions from 0.3 would indicate fewer stripes perceived compared with the Intact control stimulus.

In addition, we compared the PSE to the PTE (the SF at which the gap stimulus should appear equivalent to the control when no perceptual filling-in occurs). If the PSE is the same value as PTE, this would indicate no perceptual filling-in occurring. The PTE was calculated individually for each participant and the average was 0.49 cpd. A leftward shift in the psychometric functions from 0.49 would indicate more stripes perceived (overestimation) compared with a faithful representation of the gap stimulus.

Participants were tested in two groups. One group of participants saw the comparison stimulus with spatial frequencies of 0.25 to 0.45 cpd, whereas a second group of participants saw the comparison stimulus at 0.20 to 0.60 cpd. We were concerned the narrower range of SF for the first group may not accurately capture the PSEs of participants who do not show much perceptual filling-in and would have PSEs close to their PTE, which was on average 0.49 cpd and outside our tested stimuli range. Therefore for a second group of participants we increased the SF range up to 0.60 cpd.

**Figure 2.**
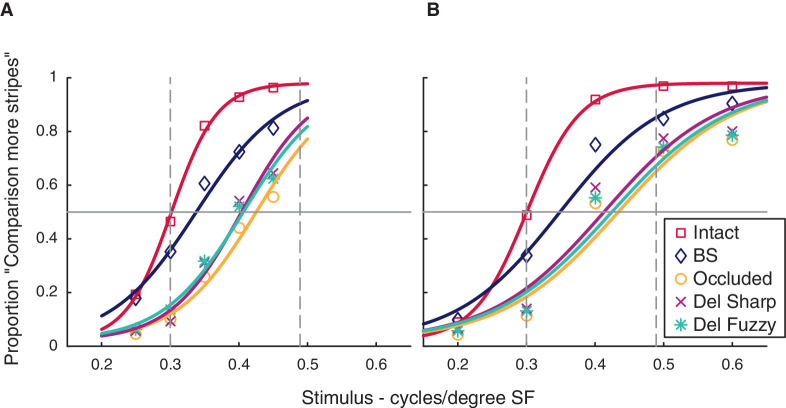
(A) Group (*n* = 37) psychometric functions for each condition. The comparison stimulus SF varied from 0.25 to 0.45 cpd. (B) Group (*n* = 23) psychometric functions for each condition. The comparison stimulus SF varied from 0.20 to 0.60 cpd. Vertical dashed line at 0.3 cpd represents the SF of the control Intact stimulus. PSEs closer to 0.3 cpd in gap conditions would indicate more overestimation of number of stripes, indicative of greater perceptual filling-in. Vertical dashed line at 0.49 represents the cpd the gap stimulus would be required to have in order to be equivalent to the Intact control stimulus (PTE, see text for explanation) averaged across participants. PSEs at 0.49 cpd would mean participants are faithfully representing the stimulus and the lack of stripes in the gap, indicative of lack of perceptual filling-in. BS = blind spot.

Figure 2 shows the group psychometric functions for each condition (all responses from all participants [except those excluded for poor blind spot calibration and fixation task performance] combined to plot a single psychometric curve for each condition). This is for visualization only, and statistical analysis was performed using values derived from individual psychometric function fits. We report data from 37 participants in the 0.25 to 0.45 cpd group, and 23 participants in the 0.20 to 0.60. Each participant completed 40 trials per each unique condition, except 2 participants in the 0.25 to 0.45 cpd group with 30 trials each (technical difficulties with data recording and noncompliance with task), and one participant who completed two blocks of 0.25 to 0.45 cpd (20 trials) and two blocks of 0.20 to 0.60 cpd (20 trials).

**Figure 3.**
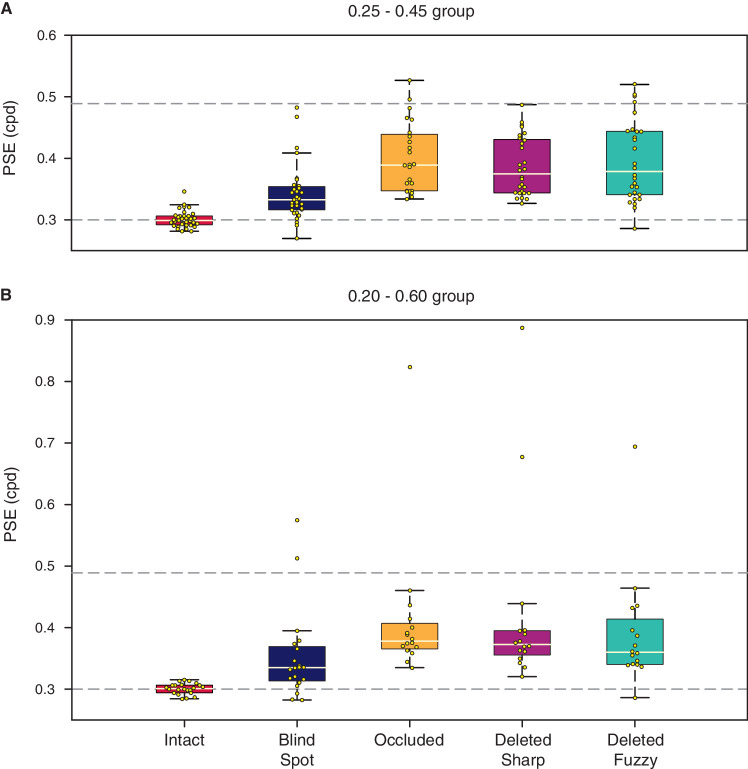
Boxplots showing the median (pale line) for PSEs in each condition. (A) PSEs for the 0.25 to 0.45 cpd group. (B) PSEs for the 0.20 to 0.60 cpd group. Horizontal dashed line at 0.3 represents the SF of the control Intact stimulus. PSEs closer to 0.3 cpd in gap conditions would indicate more overestimation of number of stripes, indicative of greater perceptual filling-in. Horizontal dashed line at 0.49 represents the cpd, the gap stimulus would be required to have in order to be equivalent to the Intact control stimulus (PTE, see text for explanation) averaged across participants. PSEs at 0.49 cpd would mean participants are faithfully representing the stimulus and the lack of stripes in the gap, indicative of lack of perceptual filling-in. Small dots show the individual data points.

The group psychometric functions suggest that PSEs for the conditions with a gap were between 0.3 cpd (maximum filling-in) and 0.49 cpd (no filling-in). To statistically compare PSEs between different conditions, we calculated the PSE separately for each participant. For the statistical comparisons we performed further data cleaning (see Methods section). Figure 3 shows the median PSEs for each condition, including the estimated PSEs for each individual participant. We performed a Wilcoxon signed-rank test to examine for significant differences between each pair of conditions, and additionally performed a paired *t*-test and calculated a Bayes factor (Table 1, Table 2, and Table A1 in the Appendix). In the text later, we describe significant differences for comparisons in which the Bayes factor suggests the evidence is in favor of the alternative hypothesis, and report *p* values (uncorrected for multiple comparisons) from the Wilcoxon signed-rank test. Whenever the Bayes factor suggests insensitivity, we err on the side of caution and do not make an inference, regardless of the *p* values.

**Table 1.** Average PSEs for each condition. SD = standard deviation; IQR = interquartile range.

	0.25–0.45 cpd				0.20–0.60 cpd			
	Median	IQR	Mean	*SD*	Median	IQR	Mean	*SD*
**Intact**	0.2989	0.0144	0.3011	0.0135	0.3010	0.0123	0.3008	0.0089
**Blind Spot**	0.3327	0.0378	0.3429	0.0467	0.3351	0.0560	0.3526	0.0727
**Occluded**	0.3890	0.0918	0.4002	0.0561	0.3782	0.0416	0.4115	0.1143
**Deleted Sharp**	0.3748	0.0869	0.3867	0.0473	0.3725	0.0397	0.4218	0.1485
**Deleted Fuzzy**	0.3786	0.1031	0.3959	0.0655	0.3600	0.0738	0.3901	0.0925

**Table 2.** Statistical comparisons between different pairs of conditions. *P* values shown are uncorrected for multiple comparisons. For ease of interpretation, statistically significant values are labeled in **bold**. For Bayes factor, values >3 are labeled as significant. Values between 0.33 and 3 suggest that the data are insensitive and are indicated using *italics.* Pairwise comparisons that we deemed to be significantly different are labeled in green; those we deemed to be equivalent are labeled in yellow. Table A1 in the Appendix reports these statistical comparisons in more detail.

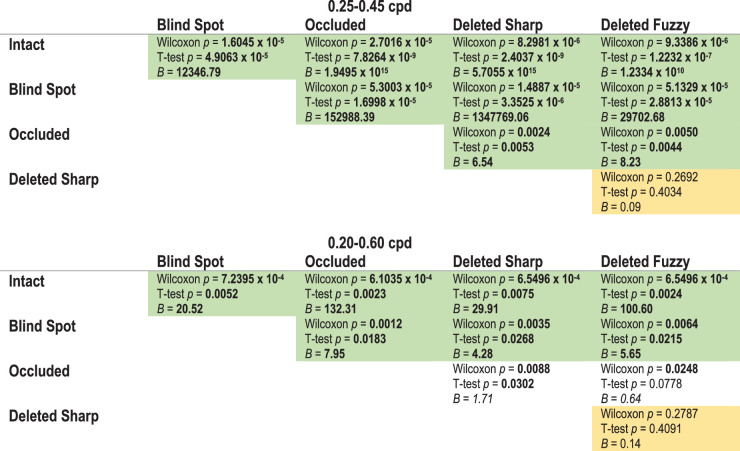

The Bayes factor calculator requires priors regarding a minimum and maximum expected effect. We subsequently report the Bayes factor as *B* (lower limit, upper limit) in the following analysis. For the PSE, we took the minimum PSE to be 0.30 (full filling-in) and maximum of 0.49 (no filling-in). The minimum and maximum difference between conditions was calculated based on these constraints. For example, comparing the Intact and Blind Spot PSE, the minimum difference would be zero (both PSEs at 0.30), and the maximum would be 0.19 (Intact at 0.30 and Blind Spot at 0.49). For the slope, we took the maximum to be the Intact slope and the minimum possible slope to be zero. We used a uniform distribution for the Bayes factor calculation.

We found that the average PSE for the Intact condition was significantly lower than for all other conditions (all *p* ≤ 7.24 × 10^−4^), suggesting none of the gap conditions led to complete perceptual filling-in. However, we found that the Blind Spot PSE was significantly lower than Occluded and Deleted conditions (all *p* ≤ 0.0064), suggesting greater perceptual filling-in in the Blind Spot compared with the artificial gap conditions. In addition, we found that the PSE in the Occluded condition was higher than Deleted Sharp and Fuzzy for the 0.25 to 0.45 group (both *p* ≤ 0.005). For the 0.20 to 0.60 group, the Bayes factor suggested that the data were insensitive for drawing conclusions on the difference between Occluded and Deleted (*B* = 1.71 and 0.64), likely because of the lower number of participants in that group. Finally, we found that PSEs were equivalent in the Deleted Sharp and Fuzzy conditions (*p* ≥ 0.2692, *B* ≤ 0.14).

### Perceptual filling-in of spatiotemporal patterns for artificial scotomata in the periphery

Overall, our results suggest that although perceptual filling-in was not complete in any condition, participants were overestimating the number of stripes in the blind spot stimulus more so than in the Occluded and Deleted stimuli. However, an outstanding question is whether participants were overestimating the number of stripes in the Occluded and Deleted conditions in relation to the veridical number of stripes in the stimulus. In other words, is there any perceptual filling-in for conditions of occlusion and deletion, or just for the blind spot? As mentioned previously, a complete lack of perceptual filling-in would result in an average PSE of 0.49 cpd. We performed paired comparisons of the average PSE and PTE of each participant to determine whether the PSE observed in the Occluded and Deleted conditions was smaller than what would be expected without perceptual filling-in.

**Figure 4.**
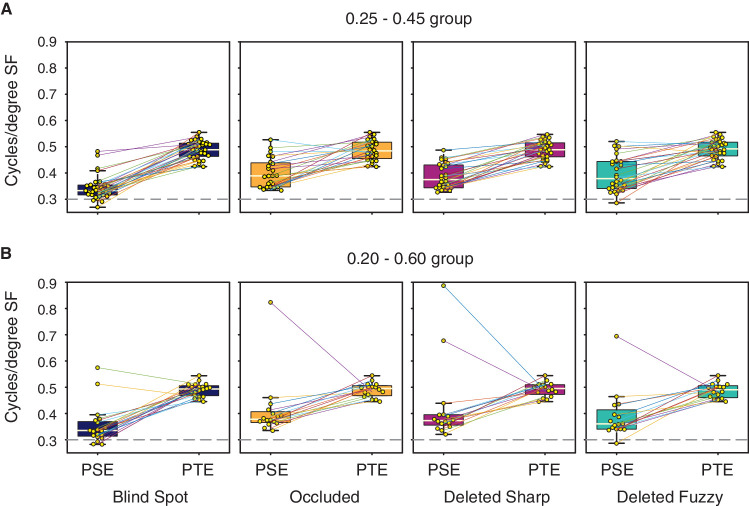
Boxplots showing the PSE versus the PTE for the (A) 0.25 to 0.45 cpd group and the (B) 0.20 to 0.60 cpd group. Pale line represents the median. Small dots represent individual data points.

Figure 4 shows the median PSE and PTE for each condition. We calculated the average bias for each condition as the difference between the PSE and the PTE (Table 3). Negative values represent the PSE being smaller than the PTE. As expected, the largest bias was observed for the Blind Spot condition. We tested for significant differences between the PSE and PTE using the Wilcoxon signed-rank test (Table 4). Additionally, we report Bayes factors for each comparison. We found that the PSE was significantly smaller than PTE for the Blind Spot (both *p* ≤ 1.63 × 10^−4^), Occluded (both *p* ≤ 0.0072), and the Deleted Fuzzy conditions (both *p* ≤ 0.0061). In addition, for the 0.25 to 0.45 cpd group of participants, PSE was significantly smaller than PTE for the Deleted Sharp condition (*p* = 3.7896 × 10^−6^), whereas for the 0.20 to 0.60 cpd group, the data were insensitive (*B* = 2.08) because of the lower number of participants in that group.

**Table 3.** Average bias (difference between PSE and PTE) for each condition. Negative values represent an overestimation of stripes. SD = standard deviation; IQR = interquartile range.

	0.25–0.45 cpd				0.20–0.60 cpd			
	Median	IQR	Mean	*SD*	Median	IQR	Mean	*SD*
**Blind Spot**	–0.1580	0.0677	–0.1455	0.0508	–0.1511	0.0964	–0.1372	0.0806
**Occluded**	–0.0882	0.0983	–0.0862	0.0634	–0.0990	0.0535	–0.0781	0.1189
**Deleted Sharp**	–0.0990	0.0600	–0.1011	0.0441	–0.1167	0.0757	–0.0695	0.1538
**Deleted Fuzzy**	–0.0985	0.0881	–0.0954	0.0651	–0.1099	0.0782	–0.0973	0.0979

**Table 4.** Statistical comparisons between PSE and PTE in each condition. *P* values shown are uncorrected for multiple comparisons. Bayes factor shown with priors as *B* (lower limit, upper limit). For ease of interpretation, statistically significant values are labeled in **bold**. For Bayes factor, values >3 are labeled as significant. Values between 0.33 and 3 suggest that the data are insensitive and are indicated using *italics.*

	0.25–0.45 cpd			0.20–0.60 cpd		
	Signed-rank test	Paired *t*-test	Bayes factor	Signed-rank test	Paired *t*-test	Bayes factor
**Blind Spot**	Z = –4.8599,	*t*(30) = –15.9387,	*B* (–0.19, 0)	Z = –3.7706,	*t*(19) = –7.6134,	*B* (–0.19, 0)
	*p* = **1.1742 × 10^−^^6^**	*p* = **3.4553 × 10^−^^16^**	= **3.9151 × 10^54^**	*p* = **1.6286 × 10^−^^4^**	*p* = **3.4697 × 10^−^^7^**	= **5.2570 × 10^10^**
**Occluded**	Z = –4.0571,	*t*(23) = –6.6637,	*B* (–0.19, 0)	Z = –2.6889,	*t*(15) = –2.6275,	*B* (–0.19, 0)
	*p* = **4.9677 × 10^−^^5^**	*p* = **8.4779 × 10^−^^7^**	= **1.7112 × 10^8^**	*p* = **0.0072**	*p* = **0.0190**	= **7.75**
**Deleted Sharp**	Z = –4.6226,	*t*(27) = –12.1435,	*B* (–0.19, 0)	Z = –1.9132,	*t*(15) = –1.8069,	*B* (–0.19, 0)
	*p* = **3.7896 × 10^−^^6^**	*p* = **1.8896 × 10^−^^12^**	= **1.1598 × 10^29^**	*p* = 0.0557	*p* = 0.0909	= *2.08*
**Deleted Fuzzy**	Z = –4.4860,	*t*(27) = –7.7466,	*B* (–0.19, 0)	Z = –2.7406,	*t*(15) = –3.9736,	*B* (–0.19, 0)
	*p* = **7.2583 × 10^−^^6^**	*p* = **2.4869 × 10^−^^8^**	= **4.6666 × 10^11^**	*p* = **0.0061**	*p* = **0.0012**	= **269.4**

Overall, results suggest that there was an overestimation of the total number of stripes in the artificial gap conditions compared with a veridical representation of the stimulus, commensurate with some perceptual filling-in occurring in the artificial scotomata.

### Additional analyses

In addition to the main analyses reported earlier, we compared the slopes of the psychometric functions (Figure A1 and Tables A2 and A3 in the Appendix). Overall, the results suggest that Intact slopes were steeper, and therefore precision in that condition was higher.

We also analyzed the reaction times for each condition (Figure A2 in the Appendix). The Blind Spot condition had the slowest reaction times, suggesting that it was hardest to judge numerosity there.

Finally, we examined the correlation between imagery strength and perceptual filling-in (Figure A3 in the Appendix), in which we found that individual differences in the average strength of perceptual filling-in were not related to each participant's general self-reported ability to visualize.

## Discussion

Previous studies have investigated perceptual filling-in in the blind spot and artificial scotoma separately, using a variety of different stimuli and placing artificial scotomata at a variety of locations in the visual field. Differences in the extent of filling-in between blind spot and artificial gaps may have been owing to differences in stimulus features and eccentricity of the stimulus. For example, filling-in of artificial scotomata improves with eccentricity (De Weerd, Desimone, & Ungerleider, 1998), and therefore perceptual filling-in may be comparable to that at the blind spot if the artificial scotoma is placed at an equally peripheral location and is of a similar size as the blind spot. A direct comparison was important, and the present study aimed to compare perceptual filling-in in the blind spot and artificial gaps of the same size and eccentricity.

We found that perceptual filling-in was stronger in the blind spot compared with an occluded or deleted section of the stimulus of corresponding size and location. This suggests that greater filling-in in the blind spot compared with artificial gaps cannot simply be attributed to its peripheral location. In addition, among the artificial gaps, we found that perceptual filling-in was slightly stronger in the Deleted conditions compared with the Occluded condition.

### Filling-in is stronger when competing feedforward information is removed

One explanation for filling-in differences between the blind spot and artificial scotomata is that filling-in might be most prominent when competing bottom-up information is removed. In the blind spot, there are no corresponding photoreceptors in the retina. Therefore this region does not receive any feedforward input, and filling-in readily occurs. This is different to an artificial scotoma in which feedforward input is available even if it is just signaling about a uniform dark surface creating a competition between representing the gap and filling it in. Our results are compatible with this explanation because we see the strongest filling-in in the blind spot in which there is least bottom-up evidence for a uniform surface at the center of the stimulus (we argue that absence of evidence for a stimulus is not the same as presence of evidence for a gap), and weakest filling-in in the Occluded stimulus in which there is strongest bottom-up evidence of a gap (the gray occluder is rather salient). Filling-in in the Deleted condition is in-between the blind spot and occluded case because although there is bottom-up stimulation of a uniform surface, the surface is the same color as the background, and thus is not as prominent as a gray occluder.

Theories of predictive coding could provide one possible explanation for this effect (e.g., Rao & Ballard, 1999). The goal of the visual system is arguably to analyze visual input for behaviorally important information. Representing whole objects despite impoverished input with spatial discontinuities is usually more useful than representing gaps. The brain optimizes perception by using sensory input to update internal models. Any discrepancy between the input and the model results in a prediction error, which is used to improve future predictions. The predictions from the internal models are based on learned statistical regularities, and would therefore signal about the completed object and bias you to perceive it as such (Shipp, 2016; Spillmann, Dresp-Langley, & Tseng, 2015). In the case of the blind spot, there is no prediction error because there is no bottom-up input, compared with the case of artificial scotoma, and thus the internal model signaling the completed stimulus has a stronger impact on the resulting percept.

Previous studies have suggested that filling-in may be aided by dampening competing feedforward signals. For example, a stroke patient experienced elongation of stimuli into the upper left visual field after nerve fibers targeting the region were affected by the stroke, thus severing input to the upper left quadrant of the visual field (Dilks, Serences, Rosenau, Yantis, & McCloskey, 2007). Loss of all bottom-up stimulation may have encouraged this elongation to develop. Similarly, in patients with age-related macular degeneration (AMD), filling-in is more likely to happen in a bilateral scotoma, and more likely in the patient's better eye (Cohen et al., 2003). The authors explain this by inputs from the better eye suppressing the worse eye and this prevents filling-in from developing. This would also explain why patients with a unilateral scotoma did not show filling-in. However, although competing feedforward stimulation may discourage development of filling-in, it is not clear if the same occurs for the blind spot, considering input from the “good” fellow eye is usually available because we view the world binocularly most of the time.

One issue with comparing cases of pathological scotomata in human patients with filling-in in the blind spot or artificial scotomata is that pathological scotomata tend to be of a different size and location. For example, macular degeneration covers a large foveal region that would affect a much larger area of cortex than a blind spot–sized lesion in the periphery, and therefore may require different mechanisms to enable filling-in, such as anatomic reorganization in the cortex. For example, it has been shown in animal studies that binocular retinal lesions lead to reorganization of the corresponding cortical circuits (Gilbert & Wiesel, 1992; Heinen & Skavenski, 1991; Kaas, Krubitzer, Chino, Langston, Polley, & Blair, 1990), but the effect is weaker for monocular lesions (Murakami, Komatsu, & Kinoshita, 1997; Schmid, Rosa, Calford, & Ambler, 1996). This could explain the results seen by Cohen et al. (2003) in which filling-in only occurred for binocular lesions, presumably because of the reorganization.

It could be argued that because the blind spot is present from birth, cortical reorganization has had time to occur, unlike in an artificial scotoma induced in an experimental session. In addition, the early onset of the scotoma resulting from the blind spot could have facilitated cortical reorganization owing to increased plasticity, unlike for retinal damage that occurs later in life, such as in AMD. However, several studies have shown that large amounts of structural reorganization are not strictly necessary for filling-in to occur. For example, Schmid et al. (1996) investigated monocular lesions and found that receptive fields around the scotoma were displaced when stimulation was through the lesioned eye, but at the same recording sites, receptive fields were unchanged when stimulation was through the normal eye. This suggests large amounts of anatomic reorganization did not occur, but rather that the lesion unmasked previously weaker effects of surrounding stimulation by silencing feedforward input. Murakami, Komatsu, and Kinoshita (1997) also did not find topographic remapping after a monocular lesion in monkeys, and perceptual filling-in occurred 2 or 3 days after the lesion. The authors argue reorganization happens after a binocular lesion but not so much after a monocular one, and that filling-in in the blind spot may not be something unique, but the same effects can be seen after a similar monocular lesion performed later in life. No irregularity or topographic remapping is seen in the natural blind spot either (Awater, Kerlin, Evans, & Tong, 2005; LeVay, Connolly, Houde, & Van Essen, 1985). Komatsu (2006) argues that reorganization is not necessary for filling-in and a different mechanism must explain filling-in at the blind spot.

Removal of competing feedforward input (bilateral lesion) seems to encourage cortical reorganization, which facilitates the development of filling-in and may be necessary for large lesions, such as retinal damage from AMD. However, filling-in is possible with monocular lesions without reorganization, suggesting a different mechanism may be involved. Dampening bottom-up input may strengthen the effect of surrounding stimulation on the silenced center of the receptive fields and/or lead to receptive field expansion, which could explain filling-in in the absence of anatomic reorganization. For example, a psychophysical study on humans showed that depriving the nonblind spot eye of any bottom-up input by having participants wear an eye-patch led to elongation of stimuli presented near the blind spot in the other eye toward the blind spot center (Dilks, Baker, Liu, & Kanwisher, 2009), similar to the stroke patient (Dilks et al., 2007). Crucially, the authors did not observe this effect when the nonblind spot eye was unpatched (but only presented with a uniform screen, not the stimulus). Dilks et al. (2009) suggest that this argues against structural differences in the cortical surface near the blind spot, and instead demonstrates rapid receptive field expansion in response to loss of feedforward input (binocular loss in the blind spot cortical representation when the nonblind spot eye is wearing the eye patch). This shows that even “meaningless” bottom-up input of a uniform surface reduces receptive field expansion, and thus diminishes filling-in. The results of our study are consistent with this idea—we found less filling-in through a dark gap viewed with the nonblind spot eye than through the blind spot. Kapadia, Gilbert and Westheimer (1994) showed results consistent with receptive field expansion when participants were presented with an artificial scotoma and showed a bias in judging the position of lines presented near the scotoma edge. However, these effects were smaller than those reported around the blind spot and may be due to bottom-up input being available and leading to weaker receptive field change (Dilks et al., 2009).

In summary, previous studies have shown that filling-in can happen after cortical reorganization, which is more likely after a bilateral lesion. For smaller or monocular lesions, large amounts of anatomic rewiring are probably not required, and this is the effect we see at the blind spot. Filling-in in such cases may occur through changes in the receptive field structure. Reducing feedforward input places more emphasis on information from stimulation in the surrounding receptive field, around the scotoma. From this we can predict that the strongest filling-in would happen through the blind spot when the opposite eye is patched, followed by blind spot viewing with the opposite eye viewing a uniform surface, and then artificial scotomata, with less salient gaps facilitating filling-in the most. Amount of filling-in in these cases could be explained by predictive coding, in which reducing bottom-up input reduces prediction error, and therefore the internal model of a completed stimulus has a greater effect on perception. Further research would be needed for a more direct test of filling-in in terms of the predictive coding framework.

### Retinal stabilization

Another potential reason why filling-in in the blind spot may be better than in artificial scotomata is retinal stabilization. The blind spot is fixed on the retina and its retinal location is not changed by eye movements. However, for artificial scotomata, even small eye movements can disrupt its retinal position and perceptual filling-in. This is especially true for filling-in via fading of the target, such as Troxler fading (Spillmann, 2011; Troxler, 1804). However, although stabilization might help, it might not be enough. For example, some studies have investigated stabilized artificial scotomata with the use of a retinal suction cap and found that although scotomata from retinal lesions filled-in instantly, artificial stabilized scotomata did not (Gerrits & Timmerman, 1969; Gerrits, De Haan, & Vendrik, 1966). We argue that stabilization differences are unlikely to fully explain the effect in the current study because we used dynamic stimuli of short duration, and therefore this is a different paradigm to studies using target fading as a measure of perceptual filling-in. Because of the dynamic nature of the stimulus, small eye movements would have disrupted the retinal stability of the spatiotemporal pattern, even in the blind spot condition.

## Conclusions

We replicate a previous study (Maus & Whitney, 2016) showing that dynamic spatiotemporal information can be filled-in across the blind spot, and this filling-in is stronger for the blind spot compared with artificial scotomata of the same size at the same eccentricity. This suggests that good perceptual filling-in at the blind spot cannot be simply attributed to its peripheral location. We propose that our results can be explained by the strength of perceptual filling-in being dependent on the amount of bottom-up evidence of a gap in the stimulus. Least bottom-up evidence exists in the blind spot, whereas an occluder of a different color to the background is the most salient, consistent with these conditions showing the most and least filling-in, respectively. This is in line with theories of predictive coding, whereby reducing feedforward input reduces prediction error, and thus perception is weighted more toward the explanation provided by predictions from the surrounding visible stimulus.

## Supplementary Material

Supplement 1
