# Supplementary material for: Stronger perceptual filling-in of spatiotemporal information in the blind spot compared with artificial gaps
Source: J Vis. 2020 Apr 28;20(4):20. doi: 10.1167/jov.20.4.20 (PMC7405704; doi:10.1167/jov.20.4.20)
Supplement: Supplement 1 [file jovi-20-4-20_s001.docx]

**Appendix**

**Table A1 | Statistical comparisons between PSEs in each pair of conditions.** *P-*values shown are uncorrected for multiple comparisons. Bayes factor shown with priors as *B*(lower limit, upper limit). For ease of interpretation, statistically significant values are labelled in **bold**. For Bayes factor, values > 3 are labelled as significant. Values between 0.33 and 3 suggest that the data are insensitive and are indicated using *italics.* Pairwise comparisons which we deemed to be significantly different are labelled in green; those we deemed to be equivalent are labelled in yellow.

|  | **0.25-0.45 cpd** | | |  | **0.20-0.60 cpd** | | |
| --- | --- | --- | --- | --- | --- | --- | --- |
| **Comparison (median, mean)** | **Signed rank test** | **Paired t-test** | **Bayes factor** | **Comparison (median, mean)** | **Signed rank test** | **Paired t-test** | **Bayes factor** |
| **Intact**  (0.3001, 0.3012)  **Blind Spot**  (0.3327, 0.3440) | Z = -4.3138,  *p* = **1.6045 x 10^-5^** | *t*(28) = -4.7913,  *p* = **4.9063 x 10^-5^** | *B*(-0.19, 0)  = **12346.79** | **Intact**  (0.3024, 0.3020)  **Blind Spot**  (0.3351, 0.3548) | Z = -3.3803,  *p* = **7.2395 x 10^-4^** | *t*(18) = -3.1758,  *p* = **0.0052** | *B*(-0.19, 0)  = **20.52** |
| **Intact**  (0.2989, 0.3024)  **Occluded**  (0.3887, 0.4006) | Z = -4.1973,  *p* = **2.7016 x 10^-5^** | *t*(22) = -9.0060,  *p* = **7.8264 x 10^-9^** | *B*(-0.19, 0)  = **1.9495 x 10^15^** | **Intact**  (0.3000, 0.2999)  **Occluded**  (0.3752, 0.4123) | Z = -3.4078,  *p* = **6.1035 x 10^-4^** | *t*(14) = -3.7254,  *p* = **0.0023** | *B*(-0.19, 0)  = **132.31** |
| **Intact**  (0.2979, 0.2996)  **Deleted Sharp**  (0.3748, 0.3854) | Z = -4.4573,  *p* = **8.2981 x 10^-6^** | *t*(25) = -9.0314,  *p* = **2.4037 x 10^-9^** | *B*(-0.19, 0)  = **5.7055 x 10^15^** | **Intact**  (0.3020, 0.3012) **Deleted Sharp**  (0.3755, 0.4259) | Z = -3.4078,  *p* = **6.5496 x 10^-4^** | *t*(14) = -3.1226,  *p* = **0.0075** | *B*(-0.19, 0)  = **29.91** |
| **Intact**  (0.2988, 0.3014) **Deleted Fuzzy**  (0.3786, 0.3954) | Z = -4.4319,  *p* = **9.3386 x 10^-6^** | *t*(25) = -7.2906,  *p* = **1.2232 x 10^-7^** | *B*(-0.19, 0)  = **1.2334 x 10^10^** | **Intact**  (0.3000, 0.3003)  **Deleted Fuzzy**  (0.3586, 0.3903) | Z = -3.4078,  *p* = **6.5496 x 10^-4^** | *t*(14) = -3.7022,  *p* = **0.0024** | *B*(-0.19, 0)  = **100.60** |
| **Blind Spot**  (0.3263, 0.3366)  **Occluded**  (0.3871, 0.3925) | Z = -4.0420,  *p* = **5.3003 x 10^-5^** | *t*(21) = -5.5387,  *p* = **1.6998 x 10^-5^** | *B*(-0.1471, 0.0429)  = **152988.39** | **Blind Spot**  (0.3350, 0.3327)  **Occluded**  (0.3811, 0.4150) | Z = -3.2374,  *p* = **0.0012** | *t*(14) = -2.6707,  *p* = **0.0183** | *B*(-0.1374, 0.0526)  = **7.95** |
| **Blind Spot**  (0.3277, 0.3385)  **Deleted Sharp**  (0.3677, 0.3826) | Z = -4.3304,  *p* = **1.4887 x 10^-5^** | *t*(25) = -5.9405,  *p* = **3.3525 x 10^-6^** | *B*(-0.1471, 0.0429)  = **1347769.06** | **Blind Spot**  (0.3335, 0.3330)  **Deleted Sharp**  (0.3725, 0.3927) | Z = -2.9191,  *p* = **0.0035** | *t*(13) = -2.4966,  *p* = **0.0268** | *B*(-0.1374, 0.0526)  = **4.28** |
| **Blind Spot**  (0.3271, 0.3371)  **Deleted Fuzzy**  (0.3669, 0.3877) | Z = -4.0495,  *p* = **5.1329 x 10^-5^** | *t*(24) = -5.1448,  *p* = **2.8813 x 10^-5^** | *B*(-0.1471, 0.0429)  = **29702.68** | **Blind Spot**  (0.3320, 0.3292)  **Deleted Fuzzy**  (0.3615, 0.3935) | Z = -2.7262,  *p* = **0.0064** | *t*(14) = -2.5879,  *p* = **0.0215** | *B*(-0.1374, 0.0526)  = **5.65** |
| **Occluded**  (0.3890, 0.3968)  **Deleted Sharp**  (0.3677, 0.3795) | Z = 3.0355,  *p* = **0.0024** | *t*(21) = 3.1067,  *p* = **0.0053** | *B*(-0.0898, 0.1002)  =**6.54** | **Occluded**  (0.3885, 0.4217)  **Deleted Sharp**  (0.3755, 0.3945) | Z = 2.6207,  *p* = **0.0088** | *t*(12) = 2.4571,  *p* = **0.0302** | *B*(-0.0785, 0.1115)  = *1.71* |
| **Occluded**  (0.3899, 0.4045)  **Deleted Fuzzy**  (0.3632, 0.3854) | Z = 2.8092,  *p* = **0.0050** | *t*(21) = 3.1892,  *p* = **0.0044** | *B*(-0.0898, 0.1002)  =**8.23** | **Occluded**  (0.3752, 0.4113) **Deleted Fuzzy**  (0.3615, 0.3925) | Z = 2.2439,  *p* = **0.0248** | *t*(14) = 1.9032,  *p* = 0.0778 | *B*(-0.0785, 0.1115)  = *0.64* |
| **Deleted Sharp**  (0.3818, 0.3899)  **Deleted Fuzzy**  (0.3786, 0.3938) | Z = -1.1049,  *p* = 0.2692 | *t*(25) = -0.8499,  *p* = 0.4034 | *B*(-0.1033, 0.0867)  =0.09 | **Deleted Sharp**  (0.3696, 0.3941)  **Deleted Fuzzy**  (0.3707, 0.4003) | Z = -1.0832,  *p* = 0.2787 | *t*(12) = -0.8553,  *p* = 0.4091 | *B*(-0.0643, 0.1257)  = 0.14 |

**Slopes**

We compared the slopes of the psychometric functions. Similar to the PSE comparisons, we removed slope values from this analysis from psychometric functions which were poor fits (see *Methods).* Figure A1 (see also Table A2) shows the median slopes in each condition. We assessed differences between conditions using the Wilcoxon signed rank test, and in addition, calculated a Bayes factor for each comparison (Table A3).


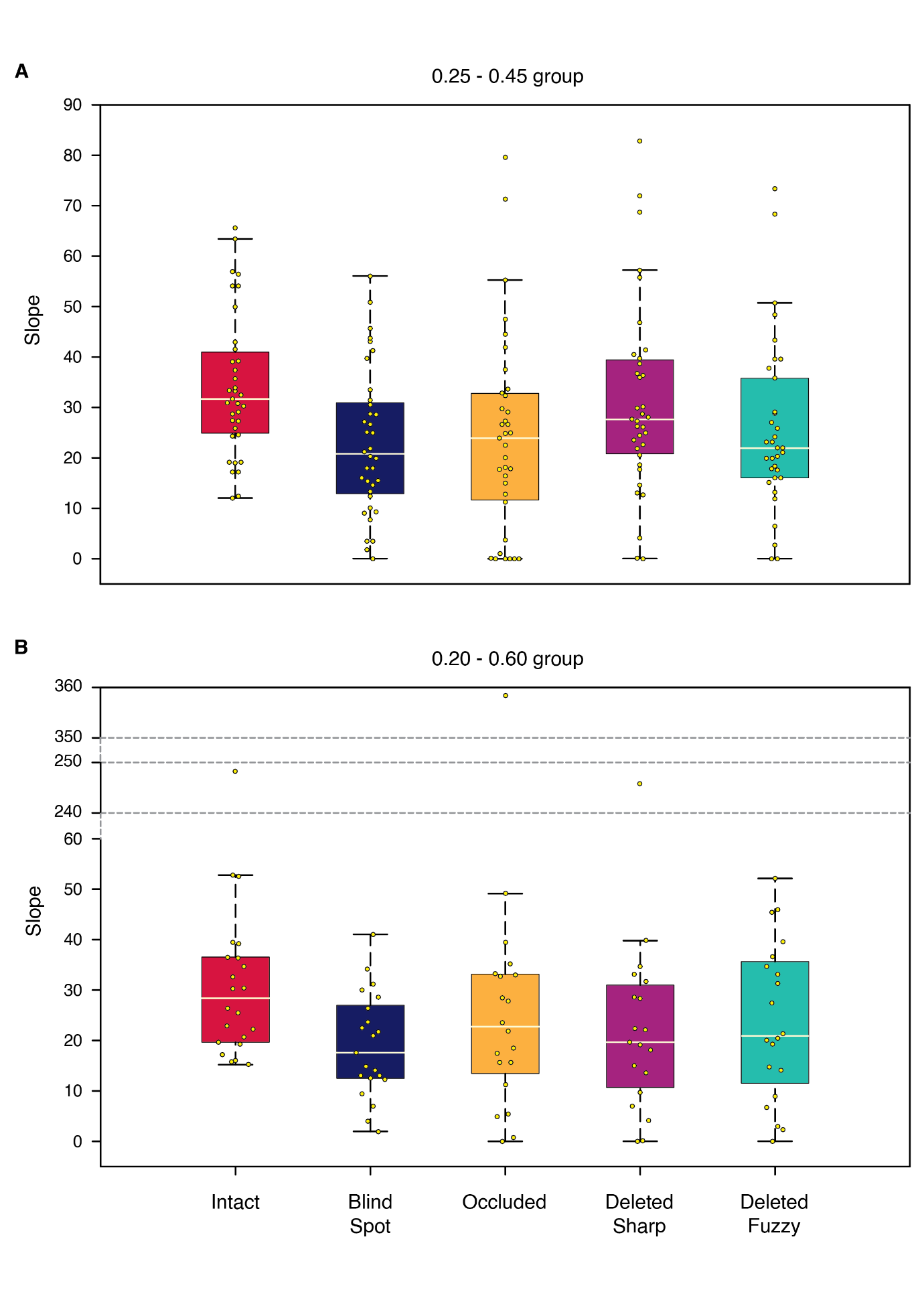


**Figure A1 | Boxplots showing the slopes of the psychometric functions** for the (A) 0.25-0.45 cpd group and the (B) 0.20-0.60 cpd group. Pale line represents the median. Small dots represent individual data points.

**Table A2 | Average slopes for each condition.** SD = Standard deviation; IQR = interquartile range.

|  | **0.25-0.45 cpd** | | | | **0.20-0.60 cpd** | | | |
| --- | --- | --- | --- | --- | --- | --- | --- | --- |
|  | **Median** | **IQR** | **Mean** | **SD** | **Median** | **IQR** | **Mean** | **SD** |
| **Intact** | 31.6794 | 16.0551 | 34.1843 | 14.1119 | 28.3671 | 16.8922 | 38.8215 | 48.0438 |
| **BS** | 20.8053 | 18.0436 | 23.0122 | 14.3328 | 17.5817 | 14.4844 | 19.0524 | 10.3151 |
| **Occluded** | 23.9105 | 21.1261 | 24.1728 | 19.5768 | 22.7338 | 19.6874 | 38.6187 | 76.4381 |
| **Deleted Sharp** | 27.6408 | 18.5838 | 31.3000 | 18.8815 | 19.6747 | 20.2851 | 31.2100 | 53.2622 |
| **Deleted Fuzzy** | 21.9291 | 19.7716 | 25.8337 | 16.8877 | 20.8989 | 24.1151 | 23.8633 | 15.6457 |

**Table A3 | Statistical comparisons between slopes in each pair of conditions.** *P-*values shown are uncorrected for multiple comparisons. Bayes factor shown with priors as *B*(lower limit, upper limit). For ease of interpretation, statistically significant values are labelled in **bold**. For Bayes factor, values > 3 are labelled as significant. Values between 0.33 and 3 suggest that the data are insensitive and are indicated using *italics.* Pairwise comparisons which we deemed to be significantly different are labelled in green; those we deemed to be equivalent are labelled in yellow.

|  | **0.25-0.45 cpd** | | |  | **0.20-0.60 cpd** | | |
| --- | --- | --- | --- | --- | --- | --- | --- |
| **Comparison (median, mean)** | **Signed rank test** | **Paired t-test** | **Bayes factor** | **Comparison (median, mean)** | **Signed rank test** | **Paired t-test** | **Bayes factor** |
| **Intact**  (31.3027, 33.7226)  **Blind Spot**  (20.1090, 22.3077) | Z = 4.0775,  *p* = **4.5519 x 10^-5^** | *t*(33) = 5.4974,  *p* = **4.2483 x 10^-6^** | *B*(0, 34.1843)  = **556138.25** | **Intact**  (25.9738, 38.2555)  **Blind Spot**  (16.2469, 18.6839) | Z = 3.5839,  *p* = **3.3845 x 10^-4^** | *t*(19) = 1.9538,  *p* = 0.0656 | *B*(0, 38.8215)  = **3.54** |
| **Intact**  (30.9260, 33.5391)  **Occluded**  (22.5123, 23.4880) | Z = 3.3324,  *p* = **8.6116 x 10^-4^** | *t*(32) = 3.8773,  *p* = **4.9371 x 10^-4^** | *B*(0, 34.1843)  = **349.40** | **Intact**  (26.4133, 39.3083)  **Occluded**  (21.8820, 21.7890) | Z = 2.3340,  *p* = **0.0196** | *t*(18) = 1.4885,  *p* = 0.1539 | *B*(0, 38.8215)  = *1.89* |
| **Intact**  (31.6794, 33.8908)  **Deleted Sharp**  (27.6408, 30.7508) | Z = 1.3312,  *p* = 0.1831 | *t*(32) = 1.1744,  *p* = 0.2489 | *B*(0, 34.1843)  = *0.34* | **Intact**  (25.5343, 39.0335)  **Deleted Sharp**  (19.1805, 31.1591) | Z = 2.2428,  *p* = **0.0249** | *t*(17) = 0.4792,  *p* = 0.6379 | *B*(0, 38.8215)  = *0.83* |
| **Intact**  (32.8519, 34.7471)  **Deleted Fuzzy**  (21.5172, 25.1775) | Z = 3.4032,  *p* = **6.6600 x 10^-4^** | *t*(31) = 4.0900,  *p* = **2.8429 x 10^-4^** | *B*(0, 34.1843)  = **735.91** | **Intact**  (28.3671, 39.4553)  **Deleted Fuzzy**  (20.8989, 22.8388) | Z = 1.7707,  *p* = 0.0766 | *t*(18) = 1.2677,  *p* = 0.2211 | *B*(0, 38.8215)  = *1.48* |
| **Blind Spot**  (20.8053, 22.4213)  **Occluded**  (24.3638, 24.2217) | Z = -0.6753,  *p* = 0.4995 | *t*(33) = -0.6981,  *p* = 0.4900 | *B*(-11.1721, 23.0122)  = 0.24 | **Blind Spot**  (17.5817, 18.7418)  **Occluded**  (21.8820, 38.5777) | Z = -1.6902,  *p* = 0.0910 | *t*(18) = -1.1377,  *p* = 0.2701 | *B*(-19.7691, 19.0524)  = *1.38* |
| **Blind Spot**  (20.8053, 23.3903)  **Deleted Sharp**  (27.8564, 31.5534) | Z = -2.6243,  *p* **= 0.0087** | *t*(33) = -2.9440,  *p* = **0.0059** | *B*(-11.1721, 23.0122)  = **13.33** | **Blind Spot**  (14.4704, 17.6688)  **Deleted Sharp**  (19.4276, 31.0177) | Z = -1.7638,  *p* = 0.0778 | *t*(17) = -1.0930,  *p* = 0.2897 | *B*(-19.7691, 19.0524)  = *0.97* |
| **Blind Spot**  (21.2441, 23.0361)  **Deleted Fuzzy**  (21.9336, 26.0142) | Z = -1.7600,  *p* = 0.0784 | *t*(32) = -1.4089,  *p* = 0.1685 | *B*(-11.1721, 23.0122)  = *0.42* | **Blind Spot**  (17.5817, 18.7929)  **Deleted Fuzzy**  (20.4524, 22.7003) | Z = -1.8914,  *p* = 0.0586 | *t*(18) = -1.3193,  *p* = 0.2036 | *B*(-19.7691, 19.0524)  = *0.44* |
| **Occluded**  (23.9105, 22.7310)  **Deleted Sharp**  (27.2940, 29.5983) | Z = -2.4870,  *p* = **0.0129** | *t*(32) = -2.6201,  *p* = **0.0133** | *B*(-10.0115, 24.1728)  = **5.26** | **Occluded**  (18.4947, 39.2356)  **Deleted Sharp**  (19.6747, 32.0405) | Z = -0.0710,  *p* = 0.9434 | *t*(16) = 0.3027,  *p* = 0.7660 | *B*(-7.601, 38.6187)  = *0.88* |
| **Occluded**  (24.3638, 24.3311)  **Deleted Fuzzy**  (21.5172, 24.2454) | Z = -0.3553,  *p* = 0.7224 | *t*(31) = 0.0441,  *p* = 0.9651 | *B*(-10.0115, 24.1728)  = 0.14 | **Occluded**  (23.5855, 39.8252)  **Deleted Fuzzy**  (21.3454, 24.3420) | Z = -0.2817,  *p* = 0.7782 | *t*(18) = 0.9305,  *p* = 0.3644 | *B*(-7.601, 38.6187)  = *1.14* |
| **Deleted Sharp**  (26.7574, 31.0290)  **Deleted Fuzzy**  (21.9291, 25.6233) | Z = 2.9544,  *p* = **0.0031** | *t*(31) = 2.6176,  *p* = **0.0136** | *B*(-2.8843, 31.3000)  = **4.66** | **Deleted Sharp**  (19.1805, 31.5410)  **Deleted Fuzzy**  (20.0450, 21.5758) | Z = -0.4971,  *p* = 0.6192 | *t*(16) = 0.8053,  *p* = 0.4324 | *B*(-7.8038, 31.0177)  = *0.97* |

The results showed that the Intact condition had a significantly steeper slope than the Blind Spot condition (both *p* ≤ 3.38 x 10^-4^). Intact slope was also higher than Occluded (*p* = 8.6 X 10^-4^) and Deleted Fuzzy (*p* = 6.66 x 10^-4^) for the 0.25-0.45 group. For the 0.20-0.60 group the data were insensitive (*B* = 1.89 and 1.48). The Intact vs Deleted Sharp comparison was also inconclusive for both groups (*B* = 0.34 and 0.83). The slope for Blind Spot was equal to the Occluded slope for the 0.25-0.45 group (*p* = 0.4995, *B* = 0.24), while for the 0.20-0.60 group this comparison was inconclusive (*B* = 1.38). The Blind Spot slope was lower than for the Deleted Sharp in the 0.25-0.45 group (*p* = 0.0087), while for the 0.20-0.60 group this comparison was inconclusive (*B* = 0.97). The Blind Spot vs Deleted Fuzzy comparison was insensitive for both groups (*B* = 0.42 and 0.44). Occluded slope was lower than Deleted Sharp slope for the 0.25-0.45 group (*p* = 0.0129), while for the 0.20-0.60 group this comparison was inconclusive (*B* = 0.88). Occluded and Deleted Fuzzy slopes were equal (*p* = 0.7224, *B* = 0.14) for the 0.25-0.45 group, while the same comparison was inconclusive for the 0.20-0.60 group (*B* = 1.14). Finally, Deleted Sharp slope was higher than Deleted Fuzzy (*p* = 0.0031) for the 0.25-0.45, while the data being insensitive in the 0.20-0.60 group (*B* = 0.97).

Overall, the results suggest that precision was better in the Intact condition compared to the conditions with a gap. For the conditions with gaps, we also found some differences, such as the Deleted Sharp slope being larger than Blind Spot, Occluded and Deleted Fuzzy, for the 0.25-0.45. A larger slope could mean higher precision of the numerosity judgement in the corresponding condition, but it could also indicate stronger perceptual filling-in. This is because participants experiencing weak filling-in rarely responded that the comparison had more stripes than the control and therefore for those conditions we obtained flat psychometric functions with small slopes. In order to assess the precision on the task while minimizing the effects of small slopes due to weak filling-in, we repeated the slopes analysis this time excluding slope values if the PSE was also excluded. In the following, we report *p-*values from the Wilcoxon sign rank test and Bayes factors. We found that Intact was higher than Blind Spot (0.25-0.45 group: *p* = 2.9246 x 10^-4^, *B* = 4507.21; 0.20-0.60 group: *p* = 5.3852 x 10^-4^, *B* = 3.16). Intact was higher than Deleted Fuzzy for the 0.25-0.45 group (*p* = 0.0043, *B* = 30.06), but equivalent in the 0.20-0.60 group (*p =* 0.4955, *B* = 0.14). Intact was also equivalent to the Deleted Sharp condition in the 0.20-0.60 group (*p* = 0.1118, *B* = 0.32). For the 0.25-0.45 group, Deleted Sharp was higher than the Blind Spot (*p =* 0.0176, *B* = 3.88) and Deleted Fuzzy (*p* = 0.0034, *B* = 21.17). For the 0.20-0.60 group, Deleted Fuzzy was higher than the Blind Spot (*p* = 0.0038, *B* = 7.02). All other comparisons were inconclusive. Overall, it appears that precision was lowest in the Blind Spot (mean = 25.63, all other conditions 29.69-34.18, for 0.25-0.45 group; mean = 19.91, all other conditions 28.94-46.41, for 0.20-0.60 group). However, due to the large number of insensitive comparisons, it is difficult to make strong conclusions about precision across conditions.

**Reaction Times**

Figure A2 shows the mean reaction times (RTs) in each condition for all participants combined across the two stimuli groups. Paired t-tests showed that participants were significantly slower in the Blind Spot (568 ms) condition compared to Intact (476 ms, *t*(58) = 4.8709, *p* = 8.9484 x 10^-6^, *B*(0, 476) = 13784.84), Deleted Sharp (495 ms, *t*(58) = 4.2337, *p* = 8.3038 x 10^-5^, *B*(-92, 384) = 613.68) and Deleted Fuzzy (490 ms, *t*(58) = 3.8267, *p* = 3.2041 x 10^-4^, *B*(-92, 384) = 120.73). In addition, participants were significantly slower in Occluded (512 ms) than Intact conditions (*t*(58) = 3.0405, *p* = 0.0035, *B*(0, 476) = 6.24). For the non-significant comparisons Bayes factor suggests that RTs were equal (all *B* ≤ 0.22), except Occluded vs Deleted Fuzzy and Occluded vs Blind Spot for which the Bayes factor suggested data were insensitive (*B*(-36, 440) = 0.66 and *B*(-92, 384) = 2.99). Overall, reaction time data suggests that the Blind Spot condition was the hardest to judge the numerosity in.


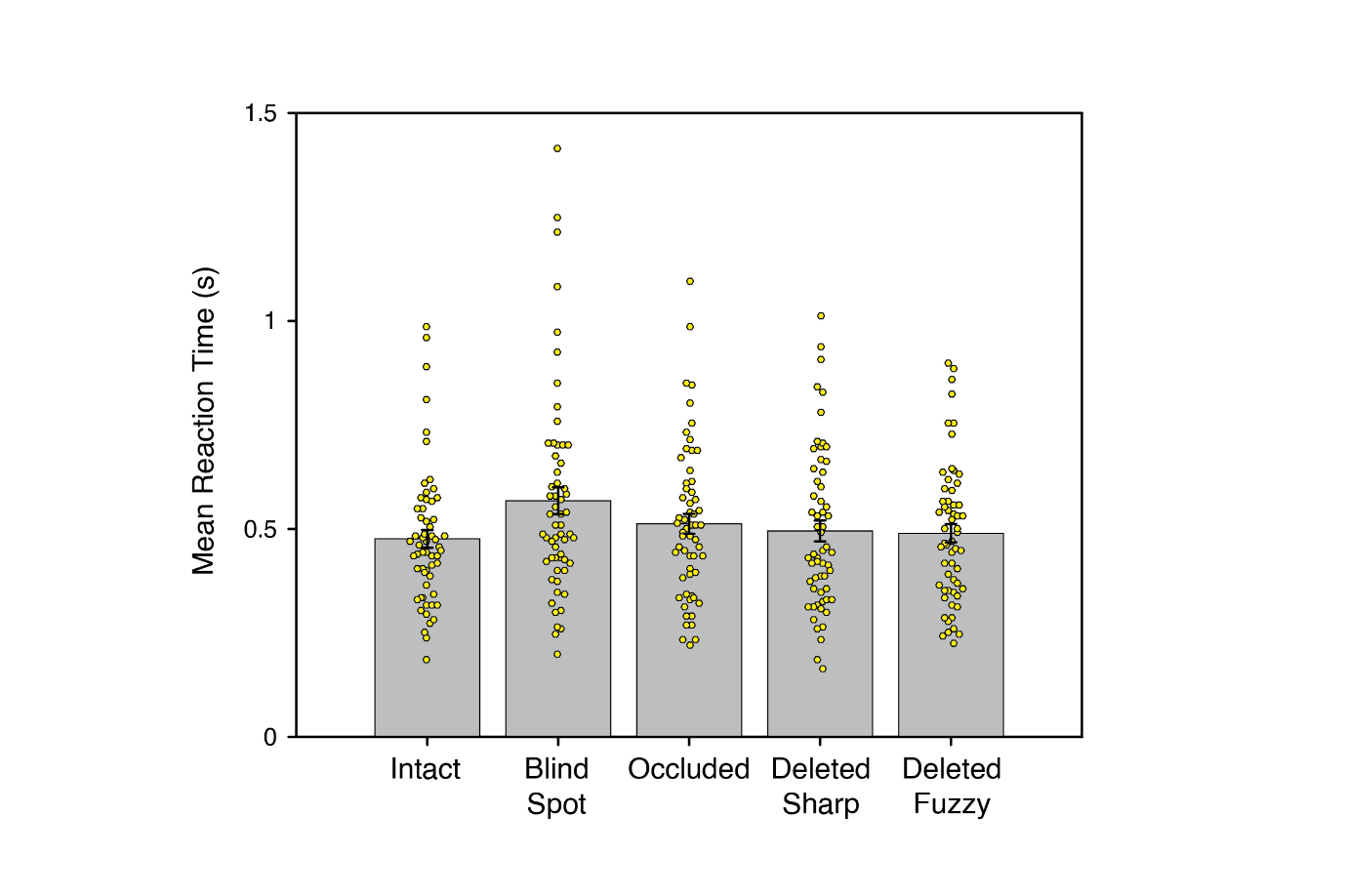


**Figure A2 | Mean reaction times (seconds) for each condition.** Error bars represent the standard error of the mean. Small dots represent individual data points.

**Vividness of Visual Imagery Questionnaire**

To assess any relationship between individual differences in visual imagery and individual differences in the strength of perceptual filling-in, we correlated the VVIQ scores (higher scores mean more vivid imagery) with the bias of the PSE for each participant (PSE – PTE, negative values mean stronger filling-in). Four participants (all in the 0.25-0.45 group) did not complete the VVIQ, and the analysis was performed on the remaining data.

We computed a Pearson’s correlation coefficient and calculated a Bayes factor for each test. Reported *p*-values are uncorrected for multiple comparisons and we deemed the significance of the correlation based on the Bayes factor. Correlation coefficients were converted to Fisher z for the calculation of the Bayes factor and we used [-6, 6] as lower and upper bounds. We did not find significant correlations for any condition (Blind Spot: N = 48, *r* = -0.2938, *p* = 0.0427, *B* = 0.24; Occluded: *n* = 36, *r* = -0.0367, *p* = 0.8316, *B* = 0.04; Deleted Sharp: *n* = 39, *r* = -0.1215, *p* = 0.4612, *B* = 0.05; Deleted Fuzzy: *n* = 41, *r* = -0.0278, *p* = 0.8628, *B* = 0.03). This suggests that individual differences in the average strength of perceptual filling-in were not related to each participant’s general self-reported ability to visualize (Figure A3).


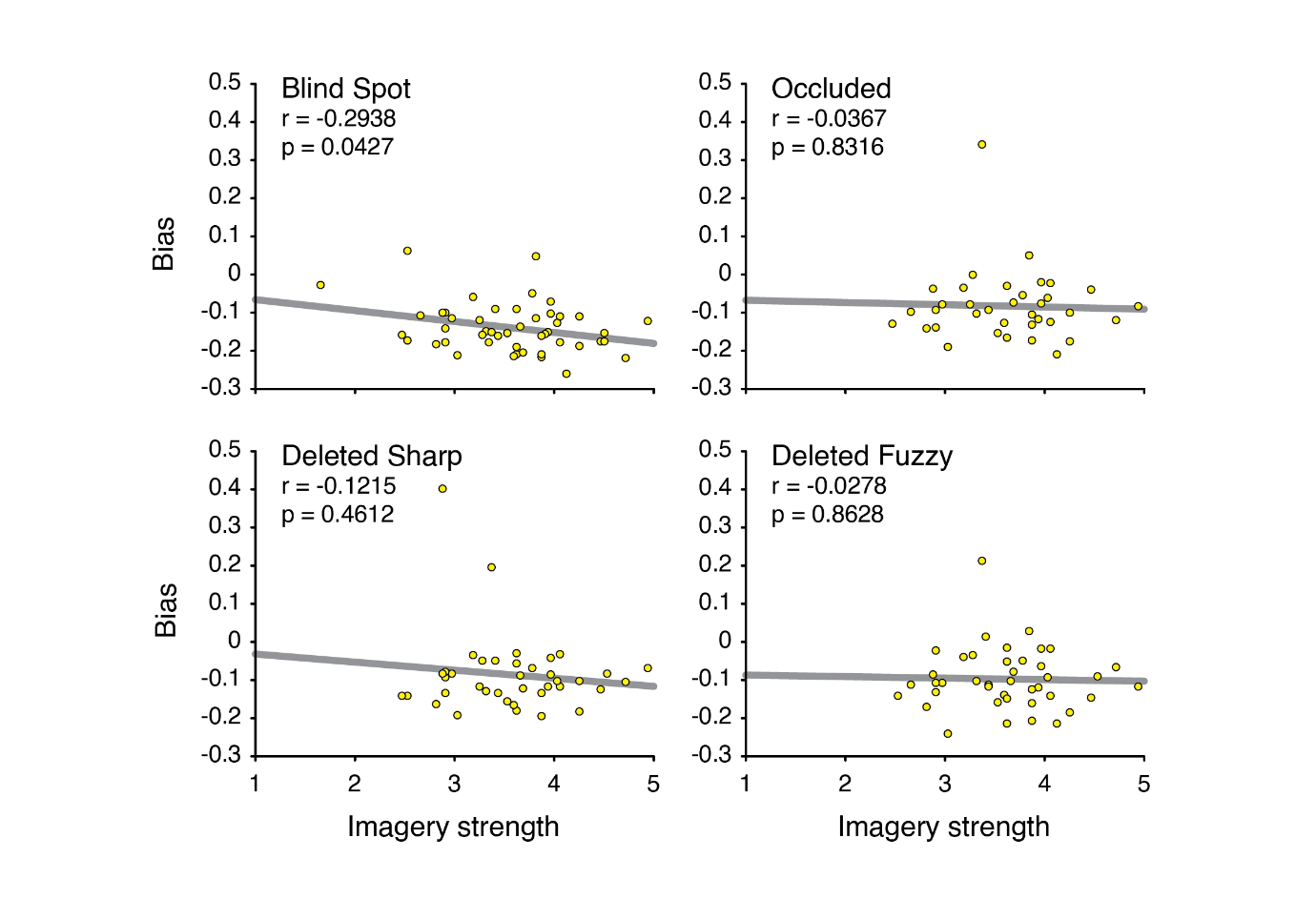


**Figure A3 | Correlation between imagery strength and perceptual filling-in.** Bias is calculated as PSE-PTE, negative values represent stronger filling-in. Higher imagery values indicate stronger imagery.
